# A Palette of Fluorescent A*β*42 Peptides Labelled at a Range of Surface-Exposed Sites

**DOI:** 10.3390/ijms23031655

**Published:** 2022-01-31

**Authors:** Dev Thacker, Mara Bless, Mohammad Barghouth, Enming Zhang, Sara Linse

**Affiliations:** 1 Department of Biochemistry and Structural Biology, Lund University, 22100 Lund, Sweden; dev.thacker@biochemistry.lu.se; 2 Department of Chemistry and Applied Biosciences, ETH Zurich, 8092 Zurich, Switzerland; blessm@student.ethz.ch; 3 Department of Clinical Sciences in Malmö, Lund University Diabetes Centre, Lund University, 22100 Lund, Sweden; mohammad.barghouth@med.lu.se (M.B.); enming.zhang@med.lu.se (E.Z.); 4 NanoLund, Lund University, P.O. Box 118, 22100 Lund, Sweden

**Keywords:** amyloid formation, optical spectroscopy, imaging

## Abstract

Fluorescence-based single molecule techniques provide important tools towards understanding the molecular mechanism of complex neurodegenerative diseases. This requires efficient covalent attachment of fluorophores. Here we create a series of cysteine mutants (S8C, Y10C, S26C, V40C, and A42C) of Aβ42, involved in Alzheimer’s disease, based on exposed positions in the fibril structure and label them with the Alexa-fluorophores using maleimide chemistry. Direct stochastic optical reconstruction microscopy imaging shows that all the labelled mutants form fibrils that can be detected by virtue of Alexa fluorescence. Aggregation assays and cryo-electron micrographs establish that the careful choice of labelling position minimizes the perturbation of the aggregation process and fibril structure. Peptides labelled at the N-terminal region, S8C and Y10C, form fibrils independently and with wild-type. Peptides labelled at the fibril core surface, S26C, V40C and A42C, form fibrils only in mixture with wild-type peptide. This can be understood on the basis of a recent fibril model, in which S26, V40 and A42 are surface exposed in two out of four monomers per fibril plane. We provide a palette of fluorescently labelled Aβ42 peptides that can be used to gain understanding of the complex mechanisms of Aβ42 self-assembly and help to develop a more targeted approach to cure the disease.

## 1. Introduction

Alzheimer’s disease is a devastating neurodegenerative disease affecting a large and growing number of individuals world-wide. Understanding the underlying molecular mechanisms is crucial to developing a targeted approach in the search for a cure. Fluorescence based super resolution microscopy methods such as direct stochastic optical reconstruction microscopy (dSTORM) or stimulated emission depletion (STED) can provide valuable structural information [1,2,3,4]. The efficient incorporation of fluorophores as molecular probes in the proteins of interest can open up avenues of additional optical methods such as for example the investigation of molecular size distributions such as fluorescence correlation spectroscopy (FCS) [5,6], cell biology using flow cytometry and fluorescence microscopy [7], Western blotting and protein interaction screening using protein arrays combined with fluorescence scanners and microarray readers [8], as well as determination of affinities and exchange rates using Förster resonance energy transfer (FRET) [9,10].

Of particular interest for neuronal function and dysfunction, fluorescence microscopy studies with fluorophore-labelled recombinant protein have provided key insights into the self assembly and membrane interaction of α-synuclein, the amyloid protein involved in Parkinson’s disease [11,12,13]. Super-resolution fluorescence microscopy studies of Aβ42, the amyloid peptide involved in the pathology of Alzheimer’s disease, have provided valuable information on the in vitro fibril elongation [14] as well as cellular uptake [15] and aggregation inside cells [16]. These studies have mainly employed synthetic fluorescent Aβ42 peptide due to challenges in successfully covalently attaching fluorophores to the peptide. Previous attempts to covalently label recombinant Aβ42 with Alexa fluorophores at the N-terminus have yielded low labeling efficiency and significantly retarded the aggregation process, which was remedied by using a low ratio of labelled to unlabelled peptide [17]. The same approach permitted the identification of glycogen synthase 3α and β (also named tau kinase 1) as interaction partners of on-pathway Aβ42 oligomers [18].

**Figure 1:**
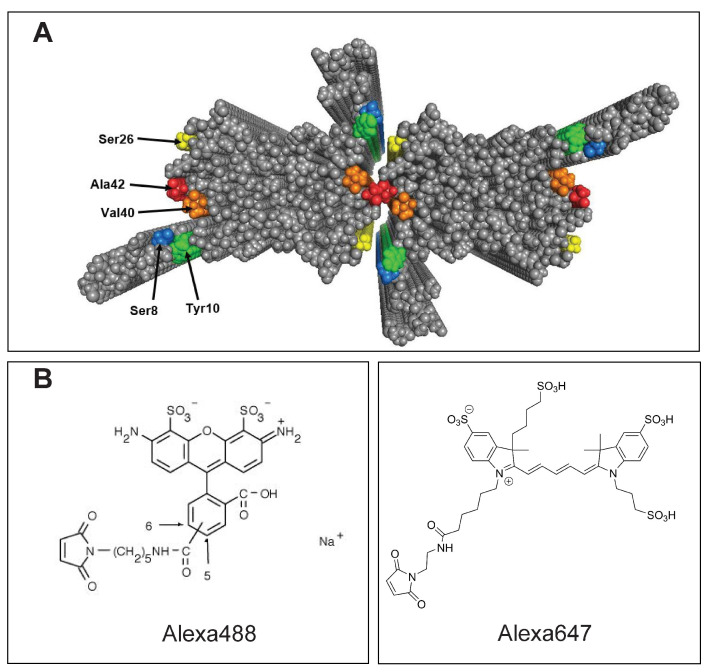
Labelling positions on the Aβ42 fibril and the fluorophore labels used. (**A**) Structure of Aβ42 fibrils with four monomers per plane based on solid state NMR [19] and Small Angle X-ray Scattering (SAXS) [21]. The positions where cysteine is introduced through site directed mutagenesis are shown—Ser8 (blue), Tyr10 (green), Ser26 (yellow), Val40 (orange), Ala42 (red). These are the positions used for covalent attachment of Alexa fluorophores. (**B**) Molecular structures of Alexa488 and Alexa647.

In the present study we optimize the position of covalent labelling of recombinant Aβ42 with fluorophores incorporated at different surface-exposed positions of the peptide in Aβ42 fibrils, as guided by recent high-resolution structures [19,20,21]. To enable covalent labelling with thiol reactive fluorophores, we thus create five cysteine mutants of Aβ42: S8C and Y10C for labelling of the unstructured N-terminal region, S26C, V40C and A42C on the surface of the fibril core (Figure 1). As a proof-of-concept, each mutant is labelled with Alexa488 and Alexa647, and the labelling efficiency is evaluated using size exclusion chromatography (SEC) and SDS PAGE. Aggregation assays of the labelled peptides, alone and in mixtures with unlabelled wild-type peptide, using thioflavin T (ThT) as well as Alexa probe fluorescence, were performed to study whether the perturbation of the fibril formation rate depends on the position of the label. Additionally, cryogenic transmission electron microscopy (cryoTEM) was used to determine the morphology of the labelled peptide fibrils compared to unlabelled WT Aβ42 fibrils.

## 2. Materials and Methods

### 2.1. Expression and Purification of Peptides

The plasmid carrying synthetic genes with *Escehrichia coli (E. coli)* optimized codons for Aβ42 wild-type (PetSac, cloned by us [22]) as well as S8C, Y10C, S26C, V40C, and A42C (Pet3a, purchased from Genscript) were transformed into Ca${}^{2+}$ competent cells of *E. coli* strain BL21 DE3 pLysS star and the protein was expressed in auto-induction medium [23]. The peptides were purified using ion exchange chromatography (IEX) as described with the minor change that lower salt concentration (50 mM NaCl, (Duchefa Biochemie, CAS no. 7647-14-5)) was used to elute the peptides, and size exclusion chromatography (SEC) on a 26 × 600 mm Superdex 75 column was used instead of spin filters for base on molecular size. The ion exchange and SEC buffers contained 1 mM dithiothreitol (DTT, PanReac Applichem, CAS no. 3483-12-3) to avoid dimerization of cysteine mutants. The final SEC was performed in buffer without DTT in order to isolate monomer and remove DTT from the sample prior to adding the label. The purified monomeric peptides were lyophilized as aliquots until further use.

### 2.2. Labelling of Purified Peptides with Alexa Fluor

Lyophilized fractions were dissolved in 50 μL milliQ water yielding a peptide concentration of ∼14 μM. Alexa fluor 488 or 647 at a concentration of 3–4 mM in 20 μL dimethyl sulfoxide (DMSO) (Sigma, CAS no. 67-68-5) was added to the dissolved peptide in order to have access dye in the labelling mixture, which was kept overnight at 4 ${}^{\circ }$C. The mixture was then added to 1 mL of 6 M GuHCl (Sigma, CAS no. 50-01-1), 20 mM sodium phosphate, 0.2 mM ethylenediaminetetraacetic acid (EDTA; J. T Baker, CAS no. 6381-92-6), pH 8.5, and subjected to SEC on a Superdex 75 10/300 column in 20 mM sodium phosphate (Merck, CAS no. 6381-92-6) buffer pH 8.0, with 0.2 mM EDTA. The absorbance at 280 nm as well as 488 nm or 647 nm was monitored using a Quadtech detector to follow the elution of the labelled peptide, excess dye as well as any unlabelled peptide, if present. The aliquots collected from the SEC were analysed by SDS PAGE, and stored at −80 ${}^{\circ }$C until further use. The same peptide concentration and conditions were used in the labelling of all mutants. Note: In case of the Y10C mutant, the absorbance at 214 nm was monitored instead of the absorbance at 280 nm. Since the Aβ42 sequence has only one Tyr residue contributing to A${}_{280}$, the Y10C mutant gives no absorbance at 280 nm wavelength. Thus, we monitor the absorbance at 214 nm, which is contributed to by all peptide bonds.

Alexa488 and Alexa647 were chosen for labelling to test labelling with chemically different probes with widely different excitation wavelengths. The peptides S8C, Y10C, and V40C were labelled with both Alexa488 and Alexa647 to enable future experiments requiring peptides labelled with two different fluorophores.

The concentration of the labelled peptides was determined using the correction factor for Alexa488 and Alexa647 in the following formula:

$$Protein\;concentration\;\left(M\right)=\frac{A_{280}-\left(A_{488/647}\cdot c.f.\right)}{\epsilon_{280}}$$

where, *A*${}_{488/647}$ is the absorbance at 488 or 647 nm, *c.f*. is a correction factor, which is 0.11 for Alexa488, and 0.03 for Alexa647, and ϵ${}_{280}$ is the extinction coefficient for Aβ42 which is 1400 L mol${}^{-1}$cm${}^{-1}$.

### 2.3. Preparation of Samples for Kinetic Experiments

The lyophilized aliquots of the purified WT Aβ42 were dissolved in 1 mL of 6 M GuHCl, 20 mM sodium phosphate, 0.2 mM EDTA, pH 8.5, and subjected to SEC on a Superdex 75 10/300 column in 20 mM sodium phosphate buffer pH 8.0, with 0.2 mM EDTA. The middle part of monomer peak was collected in a low-binding tube (Axygen, MCT-150-L-C) on ice, and was typically found to have a concentration in the range 20–80 μM (determined by absorbance of the collected fraction using ϵ${}_{280}$ = 1400 L mol${}^{-1}$cm${}^{-1}$). Frozen aliquots of the Alexa-labelled peptides were kept on ice for thawing.

### 2.4. Aggregation Kinetics by Thioflavin T Fluorescence

Aggregation kinetics experiments were performed for samples with different ratios of Alexa-labelled peptide to WT Aβ42, as follows, 1:1.5, 1:3.5, 1:7, 1:15, 1:23, 1:31 and 1:38 in 20 mM sodium phosphate and 0.2 mM EDTA, pH 8.0 buffer. The total monomer concentration was close to 5 μM. The samples were pipetted as multiple replicates into a 96-well plate (Corning 3881), 100 μL per well. The experiments were initiated by placing the 96-well plate at 37 ${}^{\circ }$C in a plate reader (Fluostar Omega). The ThT fluorescence was measured through the bottom of the plate every 120 s using an excitation filter at 440 nm and an emission filter at 480 nm.

### 2.5. Cryo-TEM

For all peptides, fibrils were prepared from samples with monomer close to 5 μM total concentration, which were incubated at 37 ${}^{\circ }$C in PEGylated plates (Corning 3881) in a plate reader and collected after reaching the plateau in ThT fluorescence. Specimens for cryo-TEM were prepared in an automatic plunge freezer system (Leica EM GP). The climate chamber temperature was kept at 21 ${}^{\circ }$C, and relative humidity was ≥90% to minimise loss of solution during sample preparation. The specimens were prepared by placing 4 μL solution on glow discharged lacey formvar carbon coated copper grids (Ted Pella) and blotted with filter paper before being plunged into liquid ethane at −183 ${}^{\circ }$C. This leads to vitrified specimens, avoiding component segmentation and rearrangement, and the formation of water crystals, thereby preserving original microstructures. The vitrified specimens were stored under liquid nitrogen until measured. A Fischione Model 2550 cryo transfer tomography holder was used to transfer the specimen into the electron microscope, JEM 2200FS, equipped with an in-column energy filter (Omega filter), which allows zero-loss imaging. The acceleration voltage was 200 kV and zero-loss images were recorded digitally with a TVIPS F416 camera using SerialEM under low dose conditions with a 10 eV energy selecting slit in place.

### 2.6. dSTORM

dSTORM samples were prepared by depositing 200 μL of 0.7 μM fibrils on a poly-Lysine coated # 1.5 (0.17 mm) glass-bottom dish (WillCo., Well, Germany). The fibrils were allowed to settle for 20 min and washed with 200 μL 20 mM sodium phosphate, 0.2 mM EDTA, pH 8.0 before loading the glass-bottom dish on the microscope. Samples were kept on ice and in the dark at all times.

dSTORM imaging was performed on the ELYRA P1 imaging system (Zeiss, Germany) which included an inverted microscope with 100X oil immerse objective lens (1.46 NA). The samples were mounted in the ZEISS level adjustable insert holder and placed on the PIEZO stage with Auto-focus adjusted. The fluorescence dyes were excited by the selected three laser lines, 488 nm, 543 nm and 633 nm respectively. Accordingly, the filter sets #4 for collection of the emission light were chosen dependent on the fluorescent dyes. For Alexa 488, the 488 nm laser line was used for excitation and the emission light filter was BP 495–550; for Alexa 546, a 543 nm laser line was used for excitation and the emission light filter was BP 570–620; for Alexa 647, a 633 nm laser line was used for excitation and the emission light filter was LP 655. The images were acquired onto a 256 × 256 pixel frame of an electron multiplying charge coupled device (EMCCD) camera (iXon DU897, Andor).

To generate the dSTORM images, the PALM processing function in the ZEN software was applied. First, the overlapping signals were discarded by using a multi-emitter model for the whole image sequences. Second, to distinguish the real signal peak, the mask size was set to 7 pixels and the ratio of signal/noise was set to 7. After the filtration, the lateral and axial drift during acquisition was corrected after reconstruction of dSTORM images by using the Drift function that was tested by a fluorescent beads as fiducial marker prior to the analysis. After drift correction, the images were proceeded by grouping function. Finally, the present dSTORM images were corrected in according to the distributions of followed parameters: photon number, precision size and first frame. To remove the unspecific background, the molecules were filtered out if the number of molecules was less than 10 in an area of 100 nm perimeter.

## 3. Results

### 3.1. Expression and Purification of Peptides

Sequence homogeneity and purity of the starting material is crucial for reproducible aggregation kinetics of peptides and its analysis. We thus expressed recombinant WT human Aβ42 as is, that is, without any tags except Met0, which is required to initiate translation, and purified from inclusion bodies using ion exchange and size exclusion steps, as described before. This mode of expression of Aβ(M1-42) relies on the peptide having low enough solubility to form inclusion bodies, which avoids the degradation in *E. coli*, a common fate of small unstructured proteins. We attempted this mode of expression and purification for all cysteine mutants and achieved good yield.

### 3.2. Covalent Labelling of Peptides with Alexa Fluors

**Figure 2:**
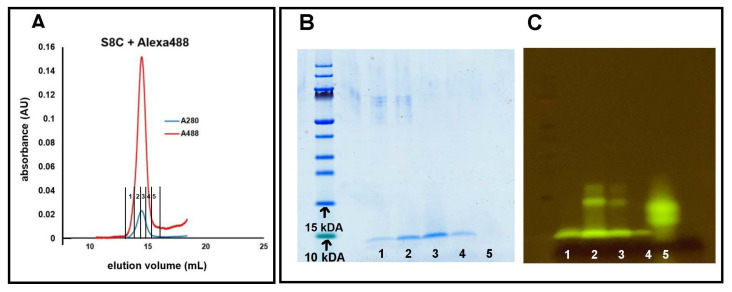
Evaluation of labelling of peptides with Alexa fluor. (**A**) Size exclusion chromatogram of S8C labelled with Alexa-488. The absorbance was monitored at 280 nm (blue) and 488 nm (red), which coincide during elution, indicating efficient labelling of S8C monomers. SDS PAGE analysis of the fractions collected from the SEC elution stained with coomassie (**B**) or with fluorescence imaging on a blue-light table with orange emission filter (**C**). Single bands prove that the monomers eluting are of the same molecular weight, and the fluorescence of the Alexa-488 dye prove that Alexa-488 has successfully bound to the S8C monomers. Note: The band at 15 kDA in wells 2 and 3 in (**C**) is a commonly observed SDS artefact for Aβ42 [22].

Peptides were labelled with Alexa fluorophores using maleimide chemistry and purified by SEC. The monitoring of the absorbance at multiple wavelengths (214 and 280 nm as well as 488 or 647 nm) indicated that all peptides are successfully labelled (Figure 2A). The eluted peptide was collected in multiple aliquots and analyzed by SDS PAGE to confirm labelling and purity. Figure 2B shows a coomassie-stained gel of eluted fractions of Alexa-488 labelled S8C. The S8C-Alexa488 monomers can be seen below the 10 kDA Mw standard in fractions 1, 2, 3, and 4. The same gel was transferred to a blue-light UV table to observe fluorescence the Alexa-488 label attached to the S8C monomers (Figure 2C). The single band for S8C-Alexa488 monomers in the coomassie-stained gel is also a strong indicator of close to ∼100% labelling of monomers. In case of inefficient labelling, an extra band with unlabelled monomers appears below the band for labelled monomers on the SDS PAGE. One such case can be seen in Supplementary Materials Figure S1D. The same labelling conditions were used for all peptides and resulted in successful covalent attachment of the Alexa fluor to the cysteine residue at the respective position in the mutant. The results can be seen in Supplementary Materials Figure S1.

### 3.3. Aggregation Kinetics

**Figure 3:**
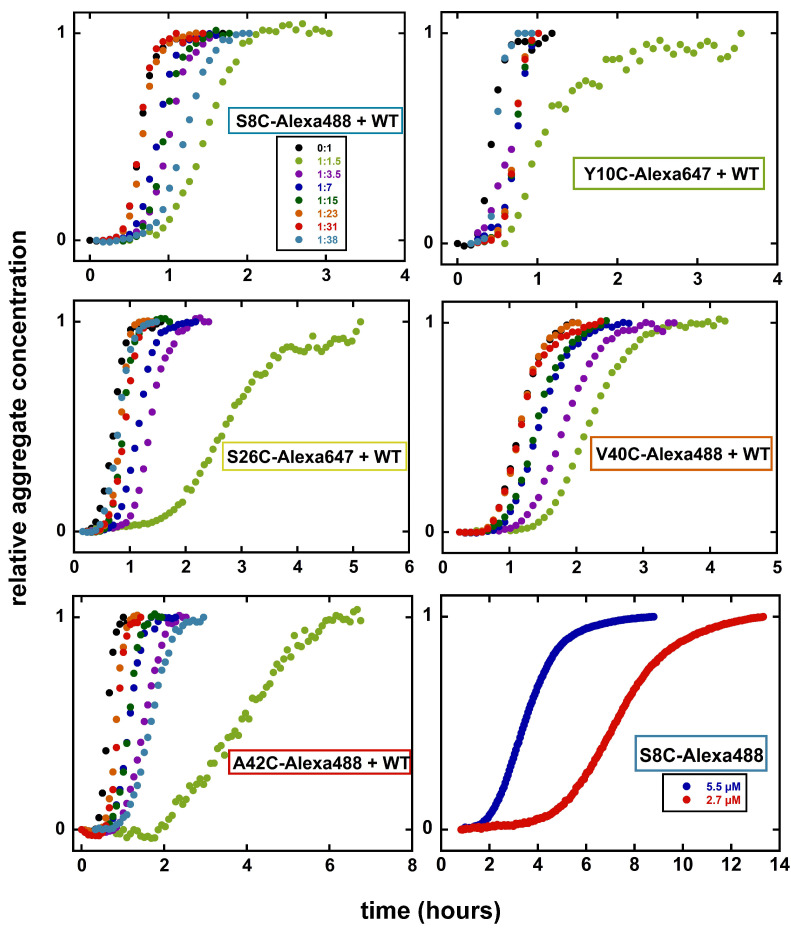
Aggregation kinetics for Alexa-labelled peptides. Aggregation kinetics for Alexa-labelled peptides in presence of different ratios of WT Aβ42, as monitored by ThT fluorescence are shown. Aggregation was monitored in the presence of 6 μM ThT in 20 mM sodium phosphate and 200 μM EDTA at pH 8.0. (color codes given in the top left panel are the same for all of the peptides). Data are the median values of three replicates at each concentration from a single experiment. The bottom right panel shows the aggregation of S8C-Alexa488 alone as followed by the fluorescence from Alexa488, which is quenched upon aggregation. Normalized data are shown in all panels.

The fibril formation of the peptides was investigated under conditions at which Aβ42 is known to aggregate rapidly. Aggregation in peptide samples with a total monomer concentration of close to 5 μM with different ratios of labelled: unlabelled peptide was followed by monitoring ThT fluorescence as a function of time at 37 ${}^{\circ }$C in 20 mM sodium phosphate and 0.2 mM EDTA, pH 8.0, for all five mutants. Under these conditions, all mutant peptides form ThT-positive aggregates over time. The aggregation curves have a sigmoidal-like appearance, comprising a lag phase, an exponential phase, and a final plateau, characteristic of nucleated polymerization reactions (see Figure 3). We find that peptides labelled at S8C can be used at 1:23–1:31 ratio of S8C:WT with no change in kinetics and at 1:3.5–1:15 ratio with a small retardation. For peptides labelled at Y10C, no effects were seen for samples with 1:3.5–1:38 Y10C:WT. For peptides labelled at S26C, no effects were seen for samples with 1:15–1:38 S26C:WT, and a small retardation at ratios 1:3.5 and 1:1.5. For peptides labelled at V40C, little effects were seen for samples with 1:31–1:38 V40C:WT, but a progressively increasing retardation at ratios 1:15–1:3.5. For peptides labelled at A42C, little effects were seen for samples with 1:3.5–1:38 A42C:WT. At 1:1.5 ratio of mutant:WT, all labelled peptides caused a delay of aggregation.

Two of the labelled peptides, S8C and Y10C, were found to form fibrils on their own, albeit at much lower rate than wt Aβ42. This is exemplified for S8C labelled with Alexa488 in Figure 3.

### 3.4. Morphology of Aggregates

**Figure 4:**
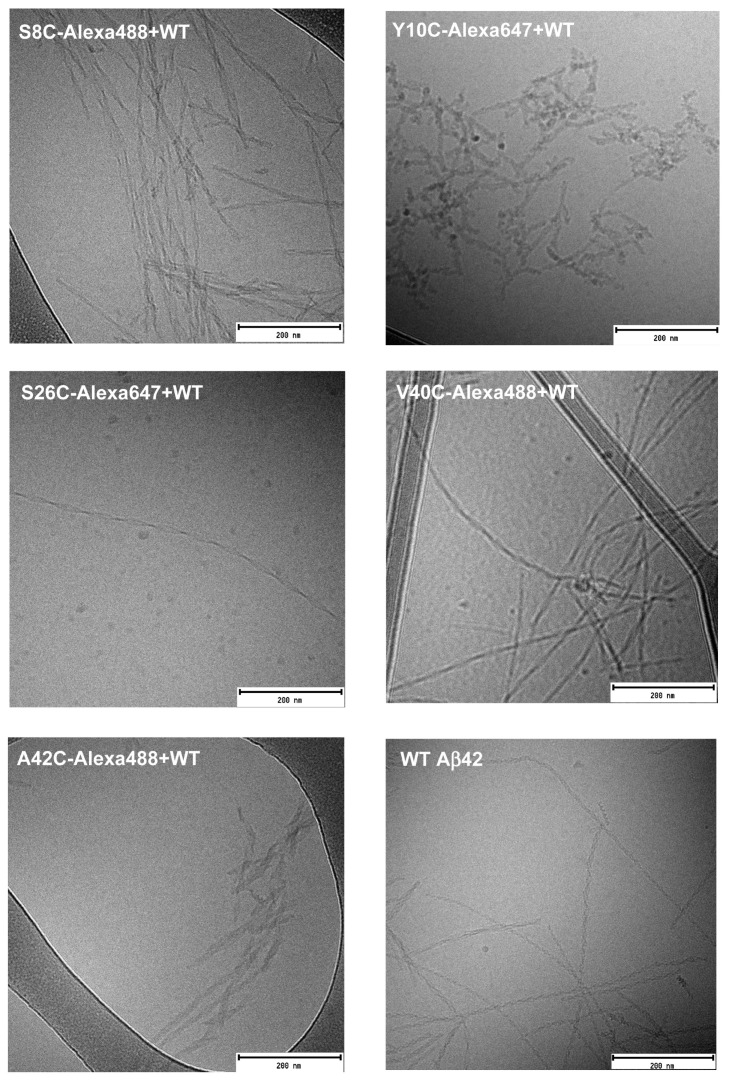
Fibril morphology by cryoTEM. Cryo-TEM of end-stage fibrils of the labelled peptides S8C, Y10C, S26C, V40C, and A42C in a 1:3.5 mixture with unlabelled WT Aβ42 are shown. A typical WT Aβ42 fibril has two filaments twisted around each other, which in a 2D image appears as nodes at regular intervals along the fibril. Fibrils formed by some of the mutants show different morphology compared to the WT peptide.

Cryo-TEM was used to study the morphology of the end-stage fibrils for all labelled peptides. In typical WT Aβ42 aggregates, individual filaments can be observed, and two filaments are twisted around each other along a common axis, seen as nodes that appear along the fibril at regular intervals. Figure 4 shows the end-stage fibrils of the Alexa-labelled peptides formed at 1:3.5 ratio with WT Aβ42. S8C-Alexa488+WT fibrils show very similar morphology to WT Aβ42. The fibrils seem rigid with sharp twists of the filaments at regular intervals. This is the same for the fibrils of V40C-Alexa488+WT, and A42C-Alexa488+WT. For Y10C-Alexa647+WT, the fibrils appear very short, and don’t appear to be as straight as the Aβ42 fibrils. The fibril morphology for S26C is also perturbed. S26C-Alexa647+WT fibrils are very long, and the node-to-node distance is longer than for typical WT Aβ42 fibrils.

**Figure 5:**
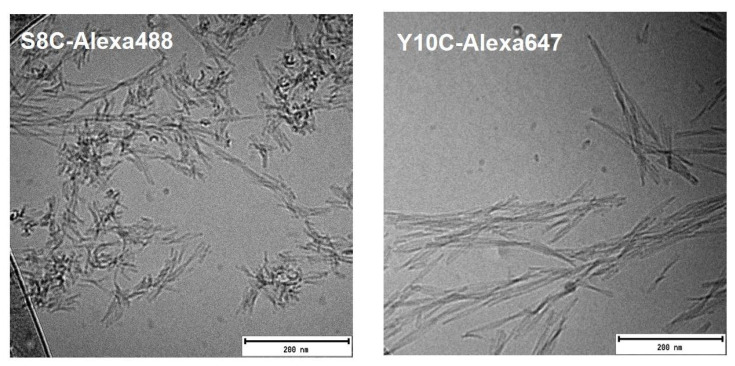
Fibril morphology by cryoTEM. Cryo-TEM of end-stage fibrils of the Alexa-labelled peptides S8C and Y10C which can aggregate without the presence of any unlabelled WT Aβ42. S8C-Alexa488 forms very short fibrils with sharp twists, while Y10C-Alexa-647 fibrils are short.

Fibrils of the two Alexa-labelled peptides formed in the absence of unlabelled WT Aβ42 are shown in Figure 5. S8C-Alexa488 forms short fibrils with sharp twists, i.e., nodes appear at short intervals. Y10C-Alexa647 fibrils are significantly shorter and exhibit a different morphology compared to the same peptide co-aggregated with WT Aβ42 as well as typical Aβ42 fibrils.

### 3.5. dSTORM Imaging of Fibrils

**Figure 6:**
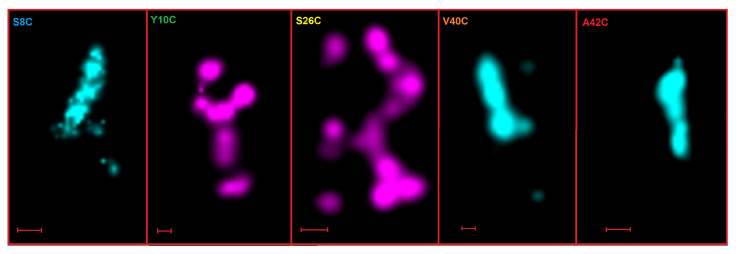
dSTORM imaging. dSTORM images of end-stage fibrils of Alexa-labelled peptides S8C-Alexa488, Y10C-Alexa647, S26C-Alexa647+WT(1:3.5), V40C-Alexa488+WT(1:3.5), A42C-Alexa488+WT(1:3.5). The scale bar represents 200 nm.

dSTORM imaging was carried out for all the labelled peptides in order to study whether the labelled peptide in incorporated in the end-stage fibrils formed in 20 mM sodium phosphate and 0.2 mM EDTA at pH 8. Figure 6 shows the dSTORM images of fibrils of the labelled peptides. The fibrils of S8C-Alexa488 and Y10C-Alexa647 which aggregate without the presence of unlabelled WT are shown, along with S26C-Alexa647, V40C-Alexa488, and A42C-Alexa488 in a 1:3.5 mix with unlabelled WT Aβ42. These dSTORM images prove that the Alexa-labelled monomers form fibrils that are detected in dSTORM microscopy by virtue of Alexa fluorescence.

## 4. Discussion

The covalent labelling of fluorophores to Aβ42 is challenging because both aggregation kinetics and fibril structure are sensitive to small changes in peptide composition and fluorophores typically have the size comparable to 7–10 amino acid residues and may be both hydrophobic and charged. Previous studies have used synthetic Aβ42 peptide for fluorescence based studies; however, it has been shown that sequence purity is of great importance in the mechanistic analysis of protein aggregation. Synthetic peptides may contain mismatches and racemates, and indeed synthetic Aβ42 is shown to aggregate significantly slower than recombinant peptide, and at the same time is unable to mimic the neurotoxic behavior of the recombinant peptide [24]. Strategic positioning of the fluorophore based on the fibril structure of Aβ42 may be one route to minimize the influence on the self-assembly of the peptide. Previous attempts to label Aβ42 with Alexa fluor at a cysteine residue added at the N-terminus resulted low labelling efficiency and caused great perturbation of aggregation kinetics. An labelled:unlabelled peptide ratio of 1:170 was required to achieve an aggregation profile similar to WT Aβ42 [17]. This might be due to the general low reactivity close to the Asp1 residue of Aβ42 peptide, as previously noted when attempting to cleave the Met0-Asp1 peptide bond [22].

In this study, we set out to find if there are more optimal fluorophore labelling positions in Aβ42 by covalently attaching Alexa fluorophores at different positions of the peptide based on their location in the fibril structure. Through site directed mutagenesis we introduce cysteine residues at five different positions of Aβ42 for fluorophore labelling using maleimide chemistry. We thus created the mutants S8C and Y10C at the N-terminal region of Aβ42, and mutants S26C, V40C, and A42C on the surface of the fibril core (Figure 1). We tested the labelling efficiency for all of these mutants, and performed time dependent aggregation kinetics for all labelled mutants in presence of WT Aβ42 in different ratios to test the extent of perturbation due to the presence of the fluorophore on the peptide. We also studied the fibril morphology and fluorophore inclusion for the end-stage fibrils of these peptides using cryoTEM and dSTORM. Whether oligomers of the labelled peptides can successfully mimic the neurotoxicity of WT Aβ42 remains to be addressed.

The eighth residue of Aβ42 is a serine, a polar amino acid on the flexible N-terminal region, which is solvent exposed in all four monomers of a fibril plane (Figure 1). We introduce cysteine, another polar amino acid, in its place through site directed mutagenesis in order to covalently attach Alex fluorophores using maleimide chemistry. The S8C mutant yields high labelling efficiency of Alexa488 as shown in Figure 1. Aggregation kinetics for S8C in presence of different ratios of WT Aβ42 show an aggregation profile very similar to WT. Even with a high ratio of labelled:unlabelled peptide (1:1.5), the lag phase is not extended significantly compared to WT alone. The fibril morphology for S8C-Alexa488+WT (1:3.5) appears to be very similar to that of typical WT Aβ42. The fibrils are straight, with two filaments twisting around each other at regular intervals creating “nodes”. Labelling Alexa-fluor at this position thus does not cause any significant perturbations of the aggregation kinetics nor fibril morphology when mixed with WT.

S8C-Alexa488 also aggregates in the absence of unlabelled WT, which was studied by following the fluorescence quenching of Alexa488 upon aggregate formation. In this case, the lag phase is considerably extended, when the aggregation of 2.7 and 5.5 μM S8C-Alexa488 is compared to 5 μM WT (Figure 3). In this case, the fibrils are much shorter than those formed in presence of an equimolar amount of WT Aβ42.

Y10C is the second mutant we created on the N-terminal region of Aβ42. We achieve high labelling efficiency in this case as well, and the aggregation kinetics don’t show any significant perturbation even at high ratio of labelled:unlabelled peptide. However, when co-aggregating with WT Aβ42, we observe that the fibril morphology is perturbed for Y10C-Alexa647. Typical WT Aβ42 fibrils appear straight and rigid. However, Y10C-Alexa647+WT (1:3.5) fibrils appear flexible and appear as short filaments uncharacteristic of mature end-stage fibrils. Y10C-Alexa647 also aggregates in the absence of unlabelled WT Aβ42. The fibrils formed from the labelled peptide alone display longer node-to-node distances but are so short that the periodical twists around the fibril length are few. Labelling Alexa-flour at this position thus seems to cause perturbation in fibril structure and morphology.

Ser26 is positioned on the surface of the fibril core of the WT Aβ42 fibril. In a fibril plane of four monomers, two Ser26 are located where the two individual filaments meet and two Ser26 appear fully exposed to water [21] (Figure 1). Replacing a hydrophilic serine residue with a large moiety such as an Alexa fluorophor could thus potentially impose steric clashes in between the filaments leading to the formation of an altered fibril morphology. At high molar ratio, 1:1.5 S26C-Alexa647:WT, this variant displays a significantly extended lag phase compared to WT alone. The fibril morphology of S26C-Alexa647+WT (1:3.5) appears to be different than that of the typical WT Aβ42 fibrils. The fibrils are longer, indicating higher elongation rate and lowered rate of secondary nucleation. The node-to-node distance is longer and the fibril diameter is larger, indicating a change in the assembly of the two filaments in the fibril. This position of labelling thus serves as an example of significant perturbation caused by the Alexa fluorophores as regards both aggregation kinetics and fibril morphology.

It has been shown that site directed mutagenesis of Val40 and Ala42 to polar serine residues does not cause any major perturbations of the aggregation mechanism and fibril morphology [25]. Hence we created mutants V40C and A42C to attach Alexa fluorophores at the respective positions. Val40 and Ala42 are also located where the two individual filaments meet (Figure 1), however unlike S26C, fluorophore-labelled V40C and A42C do not show significant perturbations of fibril morphology. As for S26C, the aggregation kinetics at high molar ratio, 1:1.5 variant:WT, is significantly perturbed, but less so or not at all at lower ratio. A possible explanation for the maintained fibril morphology at 1:3.5 molar ratio, is the possible segregation in the plane of four monomers in a fibril; one Alexa-labelled monomer may be in the positions where the labelled residues are facing the solvent, while the unlabelled WT monomers may be incorporated at the positions where Val40 and Ala42 are buried in the interface where the two filaments meet.

It is interesting to note that the labelled S26C, V40C or A42C do not seem to aggregate in the absence of unlabelled WT Aβ42. Most likely, the labelled fibrils are unable to form fibrils of an alternative fold that contains both a stable hydrophobic core and exposed fluorophores at all four positions in a fibril plane.

Finally, we note that the structures of the dye molecules Alexa488 or Alexa647 are very different, but this does not seem to affect fibril formation, which can be seen from V40C-Alexa488 and V40C-Alexa647 showing similar aggregation kinetics and fibril morphology (Supplementary Materials Figures S2 and S3).

## 5. Conclusions

In conclusion, we have successfully created a palette of different Aβ42 mutants for covalent and site-specific attachment fluorophores using maleimide chemistry. All the positions were labelled successfully and the peptides labelled at various positions can be used in different studies depending on the purpose. It is clear that the position of the fluorophore label matters for keeping the perturbation of peptide behavior to a minimum, and we find the lowest perturbation of the aggregation kinetics and fibril morphology for the labelled S8C and V40C mutants. While S8C and Y10C aggregate independently, Y10C shows altered fibril morphology when labelled with Alexa. And while V40C and A42C show little fibril morphology perturbation, they do not aggregate independently when labelled with Alexa, which can be understood based on those positions being at the interface between filaments in two out of four monomers per fibril plane. The peptides reported here may be useful in mechanistic studies of Aβ42 aggregation and cellular uptake and all plasmids will be shared with the scientific community.

## Data Availability

Data and plasmids will be shared upon reasonable request.

## Supplementary Materials

The following are available at https://www.mdpi.com/article/10.3390/ijms23031655/s1.
