# Supplementary material for: A Palette of Fluorescent Aβ42 Peptides Labelled at a Range of Surface-Exposed Sites
Source: Int J Mol Sci. 2022 Jan 31;23(3):1655. doi: 10.3390/ijms23031655 (PMC8836192; doi:10.3390/ijms23031655)
Supplement: Supplementary file 1 [file ijms-23-01655-s001.zip › ijms-1532384-supplementary.pdf]

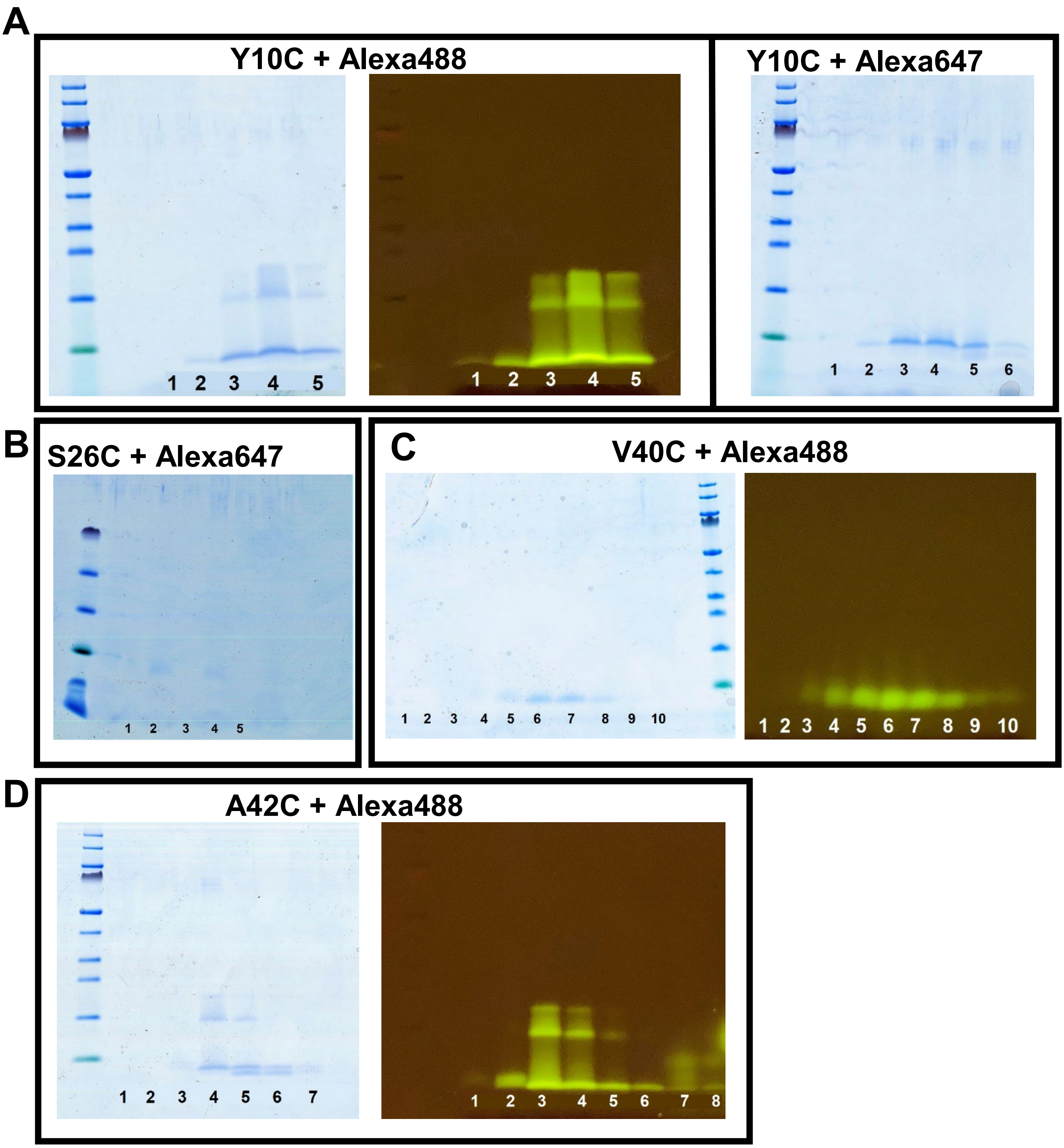

Figure S1: Covalent labelling of Alexa fluorophores to various cysteine mutants using maleimide chemistry. (A) Y10C is labelled with Alexa488 and observed on an SDS PAGE, and the same SDS PAGE gel on a UB blue light table shows fluorescence of Alexa488 on the Y10C monomer bands (left panel), and Y10C labelled with Alexa647 (right panel). (B) S26C labelled with Alexa647 yielded very low concentration resulting in faint bands on the SDS PAGE. (C) V40C labelled with Alexa488, and (D) A42C labelled with Alexa488. A fraction of unlabelled peptide can be noticed in wells 5, 6, and 7, which appears as an extra band below Alexa-labelled peptide.

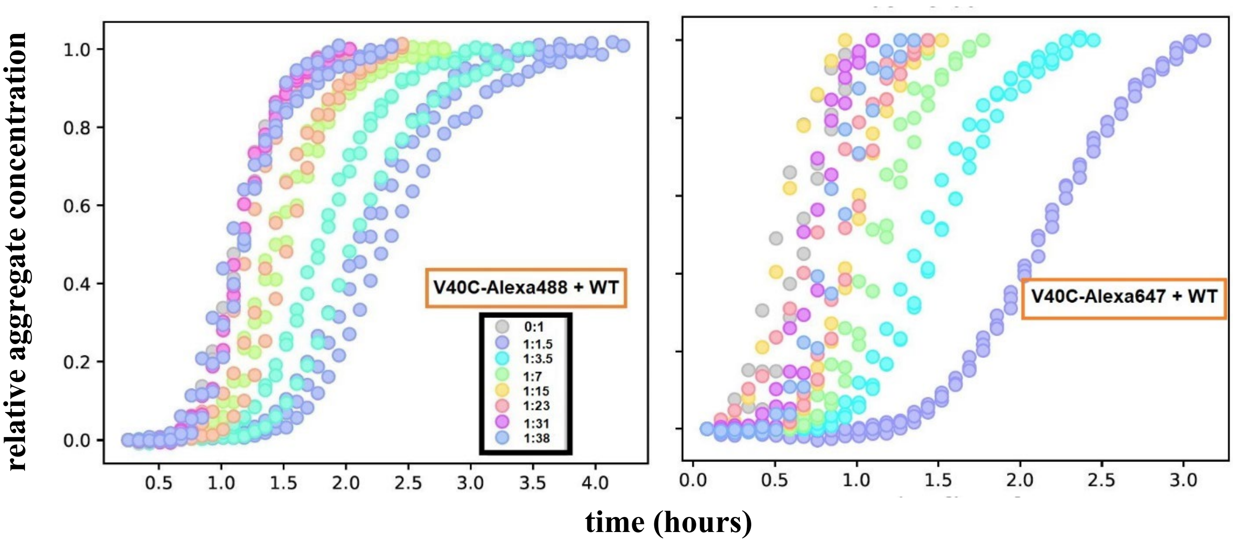

Figure S2: Aggregation kinetics of V40C-Alexa488 and V40C-Alexa647 in presence of different ratios of WT Aβ42. Total monomer concentration is close to 5 μM and time dependent aggregation kinetics are studies as a function of Thioflavin T (ThT) fluorescence in 20 mM sodium phosphate, 0.2 mM EDTA, pH 8.0 buffer in presence of 6 μM ThT. V40C-Alexa488 and V40C-Alexa647 show similar profile and time scale of aggregation, proving that both Alexa488 and Alexa647 have the same effect of low perturbation on aggregation of the peptide. Color codes given in the left panel are the same for all both the peptides.

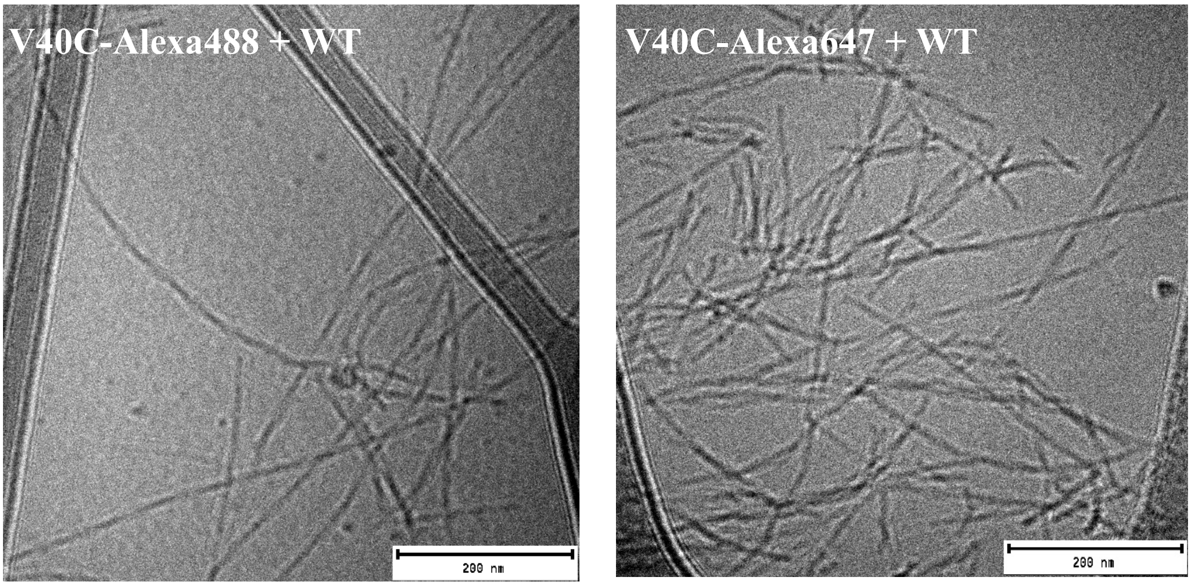

Figure S3: CryoTEM images of end-stage fibrils of V40C-Alexa488 and V40C-Alexa647 in presence of 1:3.5 molar ratio of WT Aβ42. V40C-Alexa488 and V40C-Alexa647 show similar profile fibril morphology, proving that both Alexa488 and Alexa647 have the same effect of low perturbation on fibril morphology.

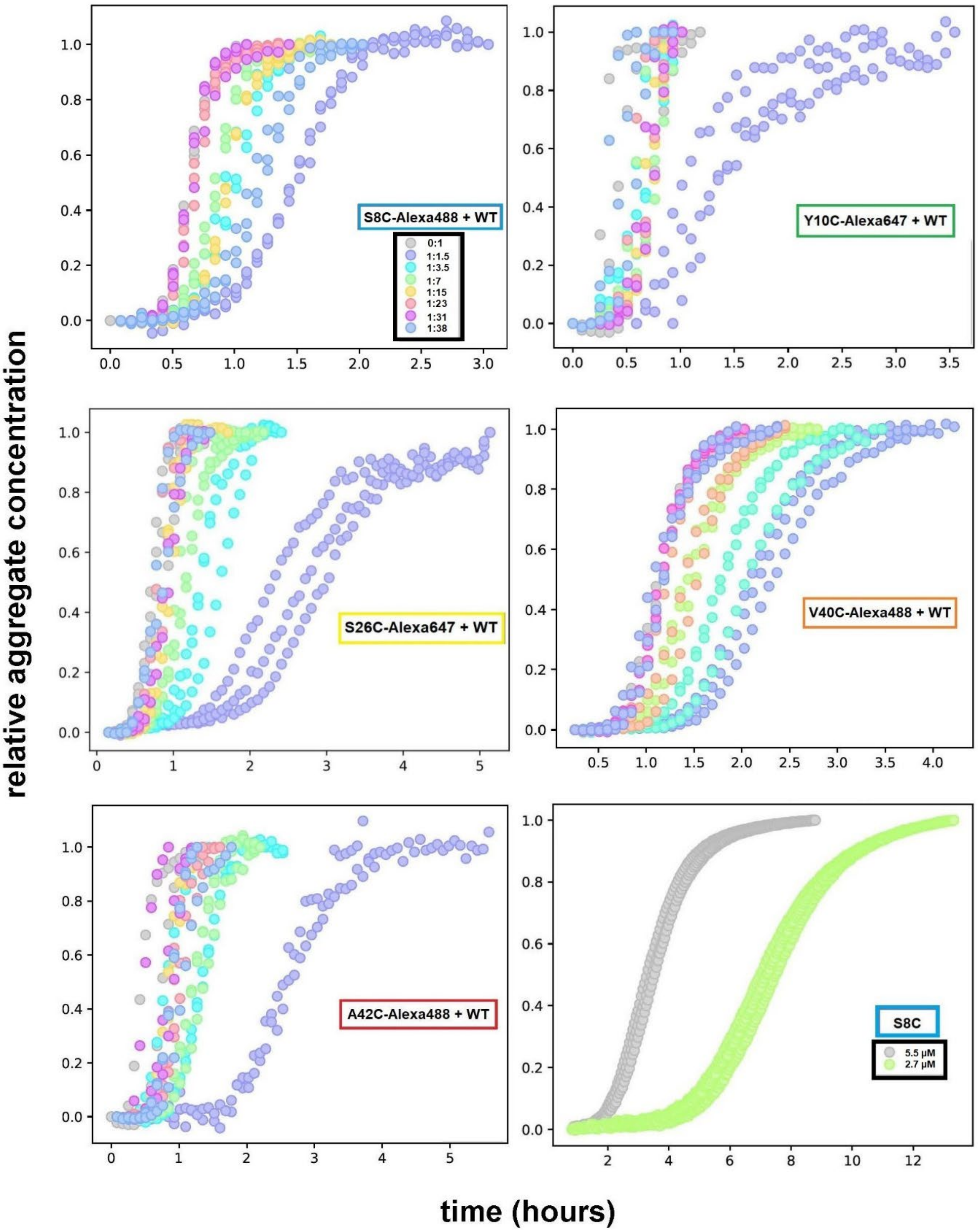

Figure S4: Aggregation kinetics for Alexa-labelled peptides in presence of different ratios of WT Aβ42, as monitored by ThT fluorescence are shown. Aggregation was monitored in the presence of 6 μM ThT in 20 mM sodium phosphate and 200 μM EDTA at pH 8.0 for samples with total monomer concentration close to 5 μM (color codes given in the top left panel are the same for all of the peptides). Data are the three replicates at each concentration from a single experiment. The bottom right panel shows the aggregation of S8C-Alexa488 alone as followed by the fluorescence from Alexa488, which is quenched upon aggregation. Normalized data are shown in all panels.

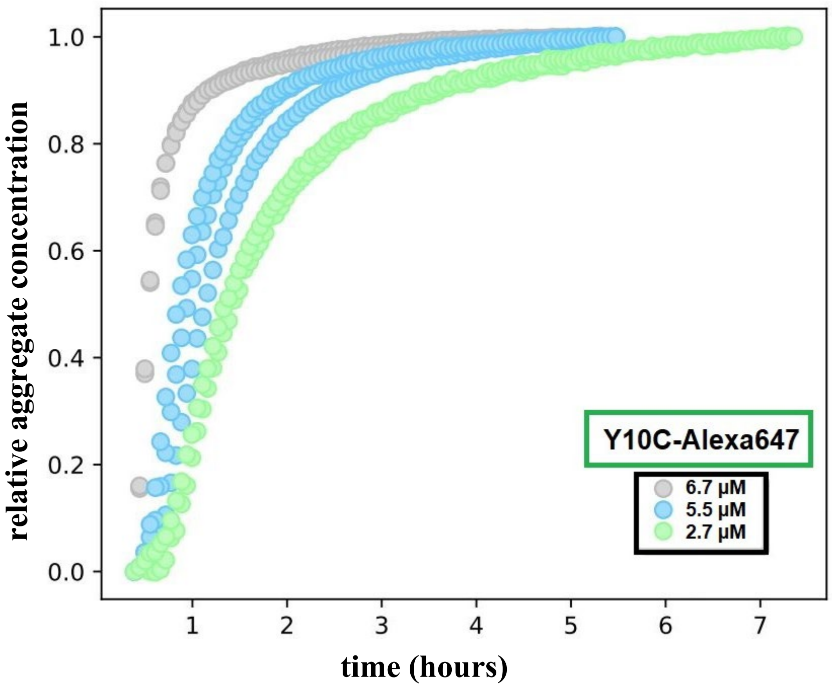

Figure S5: Aggregation kinetics of Y10C-Alexa647 in 20 mM sodium phosphate, 0.2 mM EDTA, pH 8.0 buffer at 6.7 µM (grey), 5.5 µM (blue) and 2.7 µM (green). The aggregation was followed by fluorescence from Alexa647, which gets quenched upon aggregation.
